# Square Biphasic Pulse Deep Brain Stimulation for Parkinson’s Disease: The BiP-PD Study

**DOI:** 10.3389/fnhum.2019.00368

**Published:** 2019-10-17

**Authors:** Sol De Jesus, Michael S. Okun, Kelly D. Foote, Daniel Martinez-Ramirez, Jaimie A. Roper, Chris J. Hass, Leili Shahgholi, Umer Akbar, Aparna Wagle Shukla, Robert S. Raike, Leonardo Almeida

**Affiliations:** ^1^Department of Neurology, Center for Movement Disorders and Neurorestoration, University of Florida, Gainesville, FL, United States; ^2^Department of Neurology, Penn State Milton S. Hershey Medical Center, Hershey, PA, United States; ^3^Department of Neurosurgery, Center for Movement Disorders and Neurorestoration, University of Florida, Gainesville, FL, United States; ^4^Tecnologico de Monterrey, Escuela de Medicina Ignacio A. Santos, Monterrey, Mexico; ^5^Department of Applied Physiology and Kinesiology, University of Florida, Gainesville, FL, United States; ^6^Department of Neurology, Brown University, Providence, RI, United States; ^7^Restorative Therapies Group Implantables, Research and Core Technology, Medtronic, Minneapolis, MN, United States

**Keywords:** deep brain stimulation, Parkinson’s disease, biphasic pulse stimulation, neuromodulation, therapy, pulse shape

## Abstract

**Background:**

Conventional Parkinson’s disease (PD) deep brain stimulation (DBS) utilizes a pulse with an active phase and a passive charge-balancing phase. A pulse-shaping strategy that eliminates the passive phase may be a promising approach to addressing movement disorders.

**Objectives:**

The current study assessed the safety and tolerability of square biphasic pulse shaping (sqBIP) DBS for use in PD.

**Methods:**

This small pilot safety and tolerability study compared sqBiP versus conventional DBS. Nine were enrolled. The safety and tolerability were assessed over a 3-h period on sqBiP. Friedman’s test compared blinded assessments at baseline, washout, and 30 min, 1 h, 2 h, and 3 h post sqBIP.

**Results:**

Biphasic pulses were safe and well tolerated by all participants. SqBiP performed as well as conventional DBS without significant differences in motor scores nor accelerometer or gait measures.

**Conclusion:**

Biphasic pulses were well-tolerated and provided similar benefit to conventional DBS. Further studies should address effectiveness of sqBIP in select PD patients.

## Introduction

Current FDA-approved deep brain stimulation (DBS) devices rely on stimulation delivered with traditional rectangular pulses including a stimulus pulse phase and a passive charge-balancing phase (Foutz and McIntyre, 2010; Akbar et al., 2016; Almeida et al., 2017). There may, however, be opportunities to employ other stimulation strategies to tailor therapy for individual Parkinson’s disease (PD) symptoms. Potential approaches may include the use of stimulation parameters with alternative pulse shaping (Sahin and Tie, 2007; Foutz and McIntyre, 2010; Akbar et al., 2016) or with a modification of the pulse regularity (Birdno et al., 2007, 2012).

There has been an effort to investigate alternative approaches using computer modeling (Foutz and McIntyre, 2010) and animal models (Wongsarnpigoon and Grill, 2010). Human studies have unfortunately been limited to acute testing (Birdno et al., 2007, 2012). Recently, Akbar et al. (2016) investigated the effects of novel DBS programing pulses and strategies in a study including ambulatory PD and essential tremor (ET) patients. Square biphasic pulses (sqBIP) which utilized a stimulus pulse phase and an active, rather than a passive, charge-balancing recovery phase, were associated with improvement in blinded PD motor scores, however, patients were tested for a few minutes on each condition (Akbar et al., 2016). A subsequent study by Almeida et al. (2017) demonstrated the safety and tolerability of biphasic pulses in ambulatory primary dystonia patients.

The literature is scant in regards to safety and tolerability of novel DBS strategies applied to movement disorders. Our study aimed to investigate safety and tolerability of sqBIP in PD patients in the outpatient setting for a 3 h-period.

## Materials and Methods

The protocol was approved by the University of Florida Institutional Review Board (IRB) and registered at ClinicalTrials.gov (NCT02569021). Informed consent was obtained from all participants.

### Study Participants

Patients were screened during routine DBS programing sessions conducted at the University of Florida Center for Movement Disorders and Neurorestoration. The inclusion criteria were: (1) PD diagnosis made by a fellowship-trained movement disorder neurologist using the UK brain bank criteria; (Hughes et al., 1992) (2) previously implanted DBS pulse generator compatible with available research software (Medtronic models Activa SC/PC/RC); (3) successful implantation of either globus pallidus interna (GPi) or subthalamic nucleus (STN) DBS with confirmation of a post-operative lead measurement to ensure adequate placement; and (4) patients stable on their DBS programing settings and medications and having attended a minimum of four monthly clinical programing optimization sessions (i.e., no need for further adjustments in medications or DBS settings based on response to changes performed during the prior month’s visit). The exclusion criteria were: (1) presence of alternative neurodegenerative diagnosis other than PD; (2) previously revised DBS lead (e.g., due to infection, hardware malfunction or suboptimal placement); and (3) failure to reach the minimum required number of optimization sessions or failure to achieve optimization despite multiple programing sessions.

### Study Design

**FIGURE 1:**
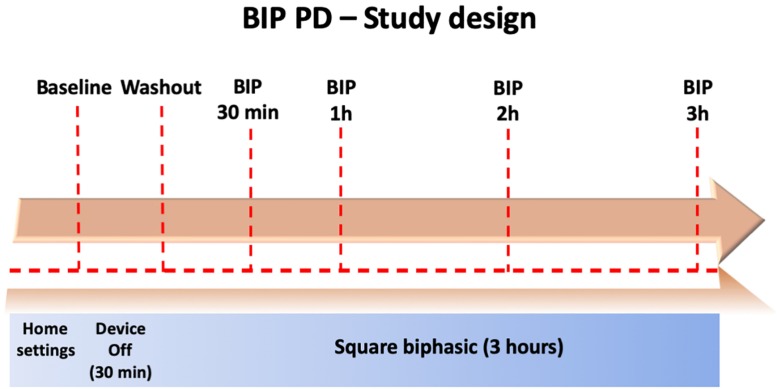
Summary of the BIP-PD study.

This was an open-label study which aimed to evaluate the safety and tolerability of sqBIP in PD. Following enrollment, patients attended a clinic visit 12 h off PD dopaminergic therapy. Patients were observed for a period of 3 h on the sqBIP setting and videotaped during serial evaluations for subsequent evaluation by raters blinded to state or time. Objective measures were obtained at condition 1 (baseline settings, ClinDBS); condition 2 (post-30 min washout with DBS off); condition 3 (30 min post-sqBIP); condition 4 (1 h post-sqBIP); condition 5 (2 h post-sqBIP); and condition 6 (3 h post-sqBIP). Figure 1 summarizes the study protocol and clinical conditions.

Each subject acted as their own control, and measurements were compared across time points. Patients with unilateral DBS were converted to sqBIP, having their motor outcomes focused on that respective hemibody under the effects of stimulation. Patients with bilateral DBS had the lead contralateral to the most affected side converted to sqBIP, and the lead corresponding to the less affected side was left unchanged, on chronic home settings, to allow comparability with patients with unilateral implants.

Subjects remained under the constant supervision of a neurologist to ensure the safety and tolerability of the experimental setting. The patients were requested to report any appearance of new symptoms or any symptom changes (worsening or improvement) during the study period, which were recorded on standardized case report forms. Each symptom change was carefully classified as to the potential relationship to the DBS status.

### Experimental sqBIP Settings and Outcome Measures

**FIGURE 2:**
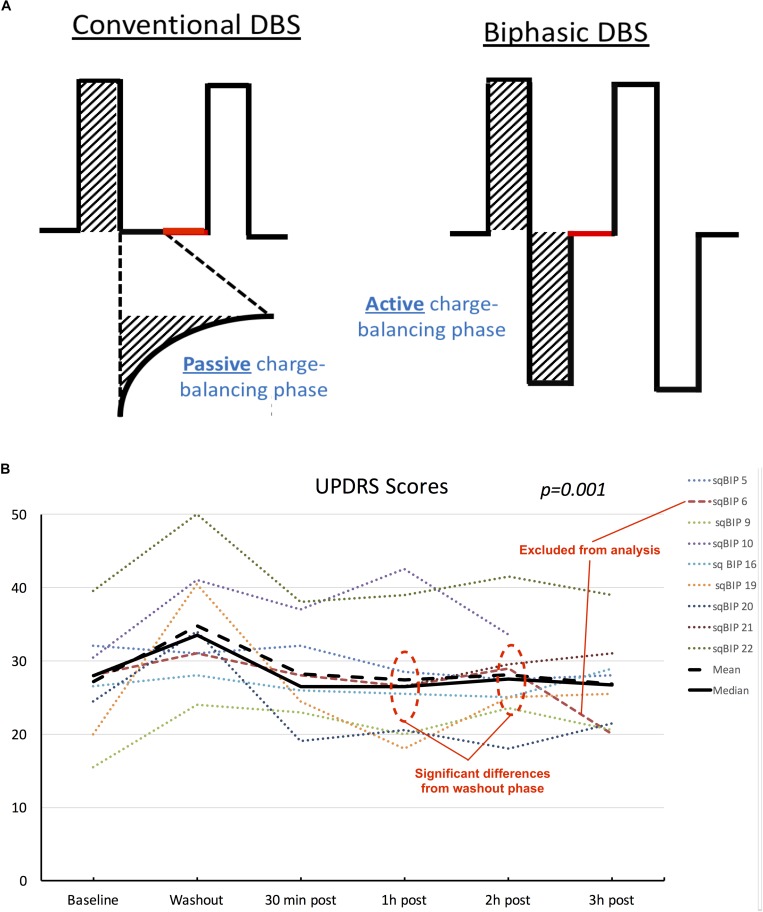
**(A)** Schematic representation of differences between conventional DBS pulse shapes (left) and sqBIP pulses (right). Stimulation parameters (amplitude, pulse width and frequency) were maintained the same from patients’ chronic home settings, similarly to intervals between pulses (represented in red). Attention is drawn to the modification in the charge balancing phase from passive to active (matching the initial depolarization phase area, as demonstrated by the dashed areas). **(B)** Individual patient scores, mean and median UPDRS values across different clinical conditions. Although data is treated as non-parametric mean values are also displayed for visual representation of skewness of the sample. Dotted lines represent individual patients, orange dashed line represents patient who was excluded from the statistical analysis. Black solid and dashed lines represent median and mean values, respectively. Statistical significance represents the overall results from the repeated measures Friedman’s test.

A charge-balanced biphasic pulse with a square-wave active recharge was used for the study. The safety standards have been previously described (Akbar et al., 2016; Almeida et al., 2017). A temporary firmware update allowed modification of the charge balancing phase to be symmetric to the discharge phase. Inter-pulse intervals and stimulation parameters (i.e., amplitude, pulse width and frequency) remained unchanged. Figure 2A exemplifies the changes in pulse shaping between conventional pulses and sqBIP pulses. The effects of sqBiP were assessed using the following objective measures: the part III of the Unified Parkinson’s Disease Rating Scale (UPDRS) motor score and accelerometer recordings (Kinesia Great Lakes NeuroTechnology, Cleveland, OH, United States) (Giuffrida et al., 2009). The GAITRite walkway (GAITRite CIR systems INC., Havertown, PA, United States) was employed to objectively measure ambulation.

### Data Analysis

The SPSS version 22.0 statistical package was utilized with a pre-defined level of 0.05 for statistical significance. The categorical variables were displayed as counts and proportions, and the continuous variables were shown as medians and interquartile ranges due to the small sample size. Repeated measures were analyzed via the non-parametric Friedman’s test and pairwise Wilcoxon test. Bonferroni corrections used for *post hoc* analysis when appropriate.

## Results

### Patient Population

Nine PD patients (5 males and 4 females) were enrolled in the study, with ages ranging from 63–75 years. The disease duration ranged from 8–25 years and the time since DBS implantation ranged from 9 months to 8.5 years. Four patients had DBS leads implanted in the STN and 5 were implanted in the GPi. One patient interrupted the study at 2 h on biphasic stimulation due to difficulties tolerating the off dopaminergic medication state, and his symptoms improved after resuming his medications. Table 1 summarizes the clinic-demographical data from the patients enrolled in the study.

**TABLE 1:** Participant clinical-demographic data.

**Subject**	**Age**	**Gender**	**Disease/DBS therapy duration**	**Unilateral vs. Bilateral DBS**	**Target tested**	**Chronic Baseline stimulation parameters**	**BIP stimulation parameters**
sqBIP 5	75	Female	15 years/10 months	Unilateral	STN	1- C + 2.3V 90PW 135Freq	1- C + 2.3V 90PW 130Freq
sqBIP 6	66	Male	11 years/4 years	Unilateral	STN	1- 2 + 3.8V 150PW 190Freq	1- 2 + 3.8V 150PW 190Freq
sqBIP 9	68	Male	20 years/11 months	Bilateral	Gpi	2- C + 2.9V 90PW 135Freq	2- C + 2.9V 90PW 130Freq
sqBIP 10	64	Female	25 years/8 years	Unilateral	Gpi	2- C + 2.2V 90PW 135Freq	2- C + 2.2V 90PW 130Freq
sqBIP 16	63	Female	8 years/9 months	Unilateral	Gpi	2- C + 1.5V 90PW 135Freq	2- C + 1.5V 90PW 130Freq
sqBIP 19	66	Male	12 years/4 years	Bilateral	STN	1- 2 + 3.1V 120PW 145Freq	1- 2 + 3.1V 120PW 145Freq
sqBIP 20	63	Male	10 years/2 years	Bilateral	STN	1-3- C + 1.9V 60PW 135Freq	1-3- C + 1.9V 60PW130Freq
sqBIP 21	75	Male	15 years/9 years	Bilateral	STN	2- C + 2.2V 90PW 135Freq	2- C + 2.2V 90PW 130Freq
sqBIP 22	72	Female	24 years/2 years	Bilateral	Gpi	3- C + 3.1V 90PW 135Freq	3- C + 3.1V 90PW 130Freq

### Safety and Tolerability

All subjects tolerated the sqBiP settings without significant adverse effects throughout the 3-h period of observation. Three patients experienced non-bothersome transient paresthesias related to device reactivation, identical to when conventional clinical settings were routinely applied during their clinical care, therefore symptoms were classified as expected programing outcomes.

### Motor Assessment

Blinded UPDRS motor scores were available for all nine patients. We excluded one patient from the statistical analysis since this patient inadvertently took the regular doses of levodopa throughout the day. Three of the patients experienced a slight improvement in the total UPDRS-III scores at 3 h post-sqBIP as compared to baseline DBS settings. The individual motor score data and variations across time are summarized in Figure 2.

Friedman’s test demonstrated statistical significance in the changes of median UPDRS motor scores values, with 26.0 (IQR = 20.5–32.5) for baseline, 34.0 (IQR = 28.0–36.5) during washout, 26.0 (IQR = 22.8–31.5), 26.0 (IQR = 19.8–28.8), 26.0 (IQR = 23.3–30.8), and 26.0 (IQR = 21.8–34.3) for 30 min, 1, 2, and 3 h post-sqBIP implementation (*p* = 0.001). Pairwise comparisons, however, demonstrated statistically significant differences only between washout and 1 h and 2 h post sqBIP, indicating potential similar effectiveness to baseline settings. A *post hoc* analysis including the patient who interrupted the study at 2 h post-sqBIP revealed similar statistical significance across the time points (*p* = 0.001).

Accelerometer data was obtained in all participants. There were no statistically significant differences between the median variables of tremor, rigidity or bradykinesia scores. Additionally, GAITRite data was available for 7 of 9 participants, and in these seven patients revealed no differences in gait speed, cadence, average step length, and average single and double support times.

## Discussion

This study demonstrated the safety and tolerability of using sqBIP pulses in a PD population over an acute 3-h study period. SqBIP provided a similar clinical benefit to conventional stimulation despite participants remaining in the off medication state. There were individual UPDRS motor scores which revealed non-significant improvement at 3 h post-sqBIP over time, and this failure to reach significance could have been due to the small sample size. Although the results point out lack of worsening in the sqBIP settings compared to regular stimulation and individual patient data may show some non-statistically significant improvement, the speculation of potential greater benefit cannot be supported by the current data and warrants further investigation with well-powered comparisons looking for efficacy. Noticeably, subject sqBIP 5 had no expected worsening during the washout phase. One may speculate that this participant might have needed a longer washout period than then the standard 30 min part of this study design, a phenomenon occasionally seen in clinical practice. There is a debate in the field on the determination of clinically significant washout periods and its potential impacts in study designs, which needs to be further explored in future studies. Additionally, the subject removed from the analysis for having mistakenly being on dopaminergic medications experienced an improvement from baseline, also reflected in his blinded UPDRS assessments (Figure 2B), suggesting a potential benefit of the addition active recharge phase in the on state comparing to conventional DBS, which should be properly addressed in a future study. Due to the lack of appropriate power analysis and estimation of effect size aiming to evaluate clinical effectiveness, the present results are restricted to safety and tolerability outcomes.

Implementation of novel stimulation strategies has been a focus of multiple DBS research groups. Modeling studies by Brocker et al. (2013) have suggested that non-regular patterns of stimulation could improve effectiveness in PD, suggesting potential applicability in clinical and experimental settings. Birdno et al. (2008) were able to modulate tremor in an already implanted DBS patient by adding intervals between pulses, suggesting that in addition to stimulation frequencies, the timing between pulses may play a role in the effectiveness of neuromodulation strategies.

Squared biphasic pulses have been tested previously in movement disorder populations. Akbar et al. (2016) tested multiple novel settings in a small sample of ET and PD patients over very small time intervals (minutes). Among all the studied settings, biphasic and shorter pulses revealed significantly improved motor scores when compared to conventional stimulation, however, being associated with a higher battery consumption (Akbar et al., 2016). More recently, Almeida et al. (2017) demonstrated the safety and tolerability of biphasic pulses in dystonia patients in a 2 h-observation study conducted in the ambulatory setting. In that study, though the sample size was small, there was a positive change in clinical response when compared to conventional DBS. Our current results are consistent with previous studies that biphasic pulses were safe and well-tolerated in movement disorders, particularly PD.

This study had several strengths as compared to previous reports. The study facilitated the testing of novel DBS programing settings by using optimized patients as their own controls and by blinding the raters to condition and time. This method reduced the possibility of several confounds that may cloud conclusions. The patients in this study were tested in the ambulatory setting for a longer observation period and this was an important procedure as the previous positive findings were reported in cases where the stimulator was only activated for a few minutes (Akbar et al., 2016). However, our data is limited to unilateral DBS testing in sqBIP mode as the initial investigative step, which may not represent “real world” data where many PD patients receive bilateral DBS. Our sample size was also small and heterogeneous in regard to presence of unilateral and bilateral implantations, as well as quite variable stimulation parameters across subjects. Therefore, although study design may allow us to have some conclusions on safety and tolerability, it clearly limits our ability to draw any conclusions on effectiveness. Patients were aware of the firmware changes in settings and though the raters were blinded, this created a potential risk for a placebo response (Mestre et al., 2016). The authors do believe, however, that the present study was an important step in replicating prior data over a period of hours, setting the ground for a future adequately powered study to evaluate efficacy of sqBIP in PD patients, as demonstrating efficacy will be crucial for a pulse shape which has been associated with a higher energy consumption.

## Conclusion

In conclusion, this study of sqBIP demonstrated its safety and tolerability over a 3-h observation period. Because sqBIP will require more energy consumption (Akbar et al., 2016), leading to more neurostimulator battery changes, patients without a response to sqBIP exceeding conventional DBS will likely not be appropriate candidates for this strategy in the future. Larger, well-powered studies will be crucial to assess symptoms responsive to this approach, and will further our understanding of sqBIP in providing alternative choices for tailored programing of individual patients.

## Data Availability Statement

All datasets generated for this study are included in the manuscript/supplementary files.

## Ethics Statement

The studies involving human participants were reviewed and approved by the University of Florida – Institutional Review Board 01. The patients/participants provided their written informed consent to participate in this study.

## Author Contributions

SD and LA were actively involved with study conception and data collection. DM-R and LS were involved with data processing, and blinded rating. JR and CH were involved with data processing and analysis. MO, KF, UA, AW, and RR were actively involved with providing expert support in different aspects of the project. All authors were involved in active manuscript preparation, and revisions, and are were in agreement with final submission.

## Conflict of Interest

RR is a paid employee of Medtronic and provides technical support but did not contribute to analysis of clinical results. MO serves as a consultant for the National Parkinson Foundation, and has received research grants from NIH, NPF, the Michael J. Fox Foundation, the Parkinson Alliance, Smallwood Foundation, the Bachmann-Strauss Foundation, the Tourette Syndrome Association, and the UF Foundation. MO’s DBS research is supported by: R01 NR014852. MO has previously received honoraria, but in the past >60 months has received no support from industry. MO has received royalties for publications with Demos, Manson, Amazon, Smashwords, Books4Patients, and Cambridge (movement disorders books). MO is an associate editor for New England Journal of Medicine Journal Watch Neurology and JAMA Neurology. MO has participated in CME and educational activities on movement disorders (in the last 36 months) sponsored by PeerView, Prime, QuantiaMD, WebMD, MedNet, Henry Stewart, and by Vanderbilt University. The institution and not MO receives grants from Medtronic, Abbvie, Allergan, and ANS/St. Jude, and the PI has no financial interest in these grants. MO has participated as a site PI and/or co-I for several NIH, foundation, and industry sponsored trials over the years but has not received honoraria. LA has received honoraria from Medtronic and Boston Scientific for consulting services and advisory board. The remaining authors declare that the research was conducted in the absence of any commercial or financial relationships that could be construed as a potential conflict of interest.
